# Diagnostic nightmare: intra-intestinal bleed masks intra-abdominal bleed after colonoscopy

**DOI:** 10.1093/jscr/rjad049

**Published:** 2023-02-22

**Authors:** Hillary M Jackson, Victoriann M Jones, Sharani U Jayaratne, Alexander A Fokin, Mohammad M Masri

**Affiliations:** Department of Graduate Medical Education, General Surgery, Larkin Community Hospital, South Miami, FL, USA; Department of Medical Education, St. George’s University, Great River, NY, USA; Department of Medical Education, St. George’s University, Great River, NY, USA; Trauma Department, Delray Medical Center, Delray Beach, FL, USA; Department of Graduate Medical Education, General Surgery, Larkin Community Hospital, South Miami, FL, USA

## Abstract

Colonoscopy is a widely used method of screening, diagnosis and intervention. Complications are infrequent and generally present as colonic perforation or colonic hemorrhage. A rare and life-threatening complication of colonoscopy is splenic injury or rupture. We present a case report of an 81-year-old female who was admitted with hemodynamic instability and tachycardia due to gastrointestinal (GI) bleeding and developed hemoperitoneum within 24 hours following colonoscopy. The initial computed tomography (CT) scan was misdiagnosed due to the patient history of GI bleed, and the iatrogenic splenic injury was recognized only during a second CT after continued hemodynamic instability. The patient’s initial diagnosis of a GI bleed masked the intraperitoneal bleed and led to a delayed diagnosis of splenic rupture and increased morbidity. This patient required an emergent laparotomy with a total splenectomy with lysis of adhesions.

## INTRODUCTION

Colonoscopy is widely used as a method of diagnosis and intervention of colonic disease with <0.1% of complications [1]. The most common complications of colonoscopy are bleeding and bowel perforation following biopsy or polypectomy [1]. One rare and underreported complication of colonoscopy is a splenic injury with intra-abdominal bleeding and hemodynamic instability, which requires a total splenectomy in severe cases [2, 3]. We present a case of splenic rupture following a colonoscopy in a geriatric female patient initially admitted for gastrointestinal (GI) bleeding.

## CASE

An 81-year-old female presented to the emergency department with a lower GI bleed and mild abdominal pain. Her past medical history included hypertension, atrial fibrillation on Eliquis, aortic stenosis, hyperlipidemia, Type II diabetes mellitus, chronic kidney disease, GERD, coronary artery stenosis, pulmonary hypertension and fibromyalgia. Past surgical history consisted of a recent trans-aortic valve replacement (TAVR), coronary stent placement, appendectomy, hysterectomy and abdominoplasty. The patient presented with a hemoglobin of 7.6 and hematocrit of 22.3. Original computed tomography (CT) showed diverticulosis and she was admitted and scheduled for colonoscopy the following day. Diverticulosis was confirmed during colonoscopy; however, no active bleed was noted. Following the procedure, the patient developed increased abdominal tenderness, tachycardia and hypotension with hemoglobin of 6.6. A CT was performed 1 hour after colonoscopy, which showed free fluid within the intraperitoneal cavity. Bowel perforation was suspected, however, free air was not visualized on CT. The patient was conservatively managed and three units of packed red blood cells were transfused, however, her vital signs continued to deteriorate. A second CT was performed 17 hours after colonoscopy, which showed increasing peritoneal fluid collection and a Grade IV splenic rupture with a surrounding hematoma and intraperitoneal hemorrhage. An emergency laparotomy with splenectomy, evacuation of intra-abdominal hematoma and extensive adhesiolysis was performed immediately following the second CT scan. The patient was discharged 12 days post-operatively.

## DISCUSSION

Splenic rupture is a rare emergency complication after colonoscopy which can lead to hemorrhagic shock if not recognized promptly. The first case of splenic rupture after colonoscopy was reported in 1974 [4]. Following the initial reported case, very few cases of this rare and devastating complication have been reported [3, 5–7]. A review of the recent literature shows that females in their 60s are most affected [5, 8–10]. Risk factors for this complication include female sex, age, adhesions from prior abdominal surgery, polypectomy, cardiovascular disease history and use of anticoagulants [1, 7, 8, 10]. The most common symptom was left upper quadrant abdominal pain [3]. Other presenting symptoms included hypotension, tachycardia and fatigue [8]. In most instances, this complication developed within 24 hours following the procedure, with some rare cases describing delayed presentations after several days [2, 5–7, 11].

In some cases, with the iatrogenic splenic injury of less severe grade, conservative management with IV fluids, blood transfusions and intensive care unit monitoring have been adequate to manage the patient [6, 7, 10, 12]. In those cases, the patient did not have severe bleeding or had a small hematoma that resolved with conservative treatment and observation. The other option in therapy for milder splenic lacerations involves splenic artery embolization to stop intra-abdominal bleeding [5–7]. Surgery was required to resolve the symptoms in most cases where the splenic rupture was Grade III and above with an accompanying large hematoma [1, 10, 12].

The definitive cause of our patient’s GI bleed is still unknown, although the patient had several risk factors, such as long-term use of Eliquis. The patient also underwent TAVR 3 months before the onset of GI bleeding. In a recent study, TAVR patients were reported as having an increased risk of intestinal bleeding in 3 months following this procedure [13].

Following literature review, there is no documented incidence of splenic rupture occurring after colonoscopy, with diagnosis initially obscured by concomitant intra-intestinal bleed. Confirmation bias is common in medicine and involves favoring information that confirms one’s original beliefs [14]. The gold standard imaging modality for the diagnosis of splenic injury is CT [15]. The most common cause of hemodynamic instability following colonoscopy is bowel perforation, which the initial CT ruled out. Due to the patient’s original presentation with GI bleed, her new onset of symptoms, which consisted of drop in hemoglobin, abdominal pain and hemodynamic instability, was considered as a continuation of the same intra-intestinal source rather than a new intra-abdominal bleeding. Consequently, the initial CT scan was misinterpreted as a worsening GI bleed, the patient was managed conservatively and surgery was not consulted. The original GI bleed masked the patient’s new intra-abdominal bleed, leading to delayed diagnosis and increased morbidity for the patient. Delayed diagnosis could also be attributed to the lack of awareness of splenic injury as a complication of colonoscopy.

Our patient possessed many risk factors for splenic injury following colonoscopy, including female sex, age, adhesions after abdominal and pelvic surgery, history of coronary artery disease and use of anticoagulation. We recommend that splenic injury be more widely recognized as a significant life-threatening complication of colonoscopy. In high-risk patients, during procedure, intracolonic pressure for bowel distension should be adjusted accordingly. Patients with an increased risk of splenic injury following colonoscopy, especially geriatric-aged female patients with previous abdominal or pelvic surgeries, should be monitored closely following the procedure and should be considered for overnight observation. Due to the possibility of delayed presentation, high-risk patients, after discharge, should be instructed to consult a physician if they experience abdominal pain and or have signs of hypotension. Abdominal pain, hypotension and tachycardia following colonoscopy should prompt a full assessment of all possible complications, including splenic injury.

## CONCLUSION

Splenic rupture following colonoscopy rarely occurs, but patients with adhesions after previous abdominal surgery as well as geriatric women and patients on anticoagulants are at risk. It is recommended that patients with the presence of risk factors are considered for overnight observation after colonoscopy. Surgeons should also be aware of the possibility of consecutive double bleeding from different sources masking each other and must suspect and address the main cause of hemodynamic instability rapidly.

## Data Availability

Data supporting the conclusions are included in the article.
